# The Role of Ferroptosis in Acute Kidney Injury

**DOI:** 10.3389/fmolb.2022.951275

**Published:** 2022-06-30

**Authors:** Jinshi Zhang, Binqi Wang, Shizhu Yuan, Qiang He, Juan Jin

**Affiliations:** ^1^ Urology & Nephrology Center, Department of Nephrology, Zhejiang Provincial People’s Hospital (Affiliated People’s Hospital, Hangzhou Medical College), Hangzhou, China; ^2^ Zhejiang Chinese Medical University, The Second School of Clinical Medical, Hangzhou, China

**Keywords:** acute kidney injury, ferroptosis, mechanism, treatment, regulators

## Abstract

Ferroptosis is a novel cell death method discovered in recent years. It is usually accompanied by massive accumulations of iron and lipid peroxidation during cell death. Recent studies have shown that ferroptosis is closely associated with the pathophysiological processes of many diseases, such as tumors, neurological diseases, localized ischemia-reperfusion injury, kidney injury, and hematological diseases. How to intervene in the incidence and development of associated diseases by regulating the ferroptosis of cells has become a hot topic of research. This article provides a review of the role of ferroptosis in the pathogenesis and potential treatment of acute kidney injury.

## Introduction

Acute kidney injury (AKI), is a disease that can have a variety of causes, including ischemia, nephrotoxic drugs, and urinary tract obstruction (Wang and Tao, 2015). AKI has high morbidity and mortality rates in hospitalized patients, and yet research on the therapeutic options for AKI prevention and treatment, other than hemodialysis, has been slow. Therefore, new therapeutic options are urgently needed to prevent AKI, as well as to promote kidney repair after AKI onset. In 2012, Dixon et al. (2012) proposed a new concept of cell death, known as ferroptosis, which was subsequently proven to be closely associated with the pathophysiological processes of many diseases (Alvarez et al., 2017; Tang et al., 2021). A recent study has shed light on the role of iron homeostasis in the pathogenesis of AKI and its therapeutic potential (Swaminathan, 2018). This article reviews the current research on the regulatory mechanisms, research progress, and therapeutic potential of ferroptosis in AKI.

## Ferroptosis

### Overview

**FIGURE 1:**
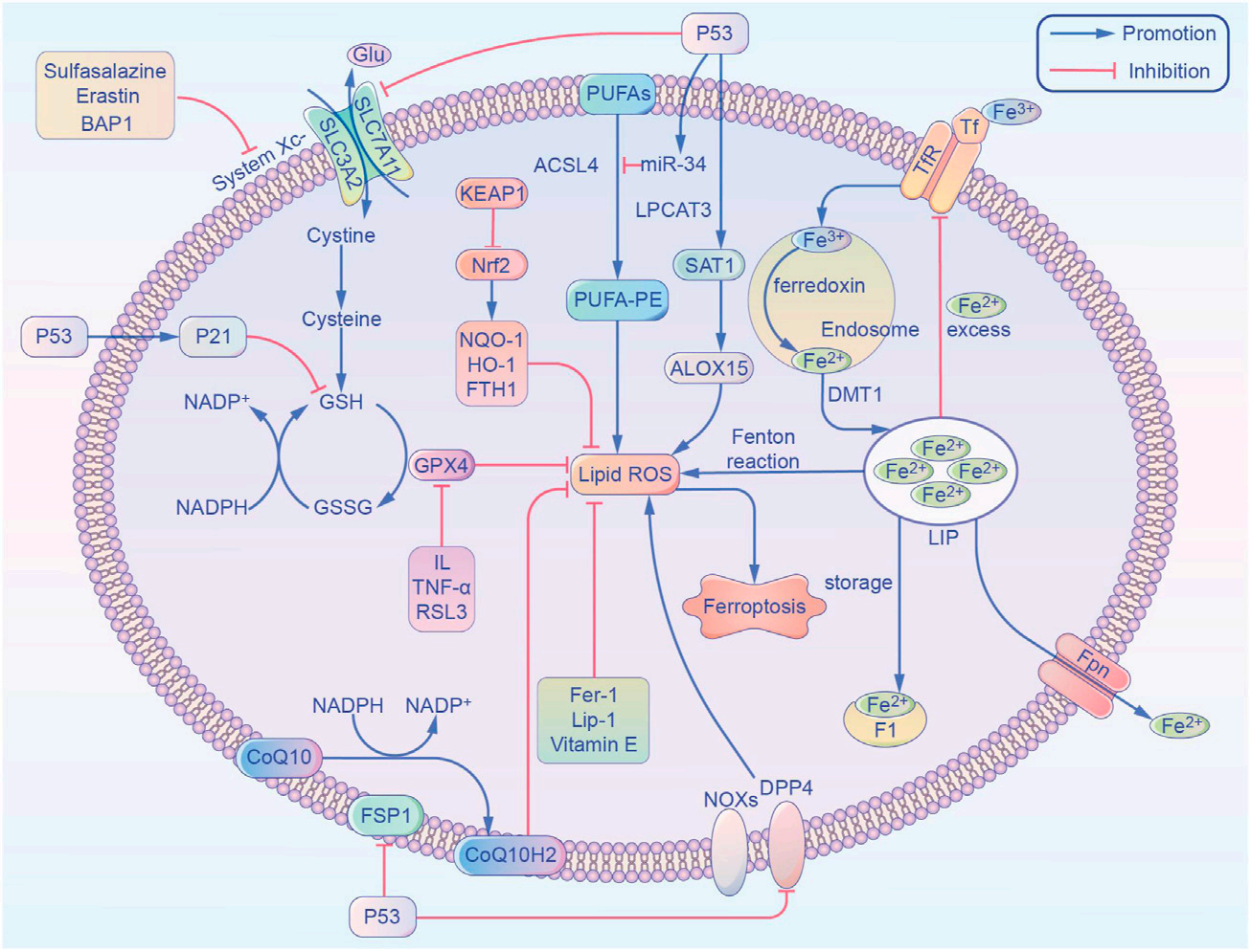
Overview of the metabolic routes contributing to ferroptosis. This non-exhaustive list includes (1) iron-Fenton reaction. (2) GPX4 antioxidant activity. (3) Lipid metabolism pathway. ACSL4, acyl-coenzyme A synthetase long chain family member 4; ALOX15, arachidonate 15-lipoxygenase; BAP1, BRCA1 associated protein 1; CoQ10, coenzyme Q10; CoQ10 H2, ubiquinol; DPP4, dipeptidyl-peptidase-4; DMT1, divalent metal transporter 1; FTH1, ferritin heavy chain 1; Fer-1, ferrostatin-1; FSP1, ferroptosis suppressor protein 1; Fpn, ferroportin; GPX4, glutathione peroxidase 4; GSH, glutathione; GSSG, oxidized glutathione; Glu, glutamic acid; HO-1, heme oxygenase-1; IL, interleukin; Lip-1, liproxstatin-1; LPCAT3, lysophosphatidylcholine acyltransferase 3; LIP, labile iron pool; NADPH, nicotinamide adenine dinucleotide phosphate; Nrf2, nuclear factor E2 related factor 2; NOX1, NADPH Oxidase 1; NQO-1, NAD(P)H quinone dehydrogenase 1; PUFAs, polyunsaturated fatty acids; ROS, reactive oxygen species; SAT1, spermidine/spermine N1-acetyltransferase 1; System Xc-, glutamate-cystine/antiporter; SLC7A11, solute carrier family 7 member 11; SLC3A2, solute carrier family 3 member 2; TNF-α, tumor necrosis factor-α; TfR, transferrin receptor; Tf, transferrin.

As one of the most important essential trace elements, iron is involved in a wide variety of metabolic processes in the body. In 2003, Dolma et al. (Yao et al., 2021) discovered a novel erastin compound that is selectively lethal to tumor cells with the RAS gene mutation, but this causes cell death in a manner that is different from conventional apoptosis without nuclear morphological changes. Neither DNA fragmentation, cysteine-containing aspartate protein hydrolase (caspase) activation, nor caspase inhibitors inhibit this mode of cell death. Subsequently, Yang et al. (Heintzman et al., 2022) and Yagoda et al. (2007) found that this mode of cell death could be inhibited by iron chelators and discovered another compound, RSL3, which could also cause this mode of cell death. In 2012, Dixon et al. (2012) officially named this mode of cell death “ferroptosis,” or “iron death,” based on its characteristics. Unlike other regulated cell deaths like apoptosis and autophagy, ferroptosis has the following characteristics: 1) Morphologically, it is primarily characterized by cell membrane rupture and blebbing, reduction in mitochondrial volume, increase in membrane density, and reduction or even disappearance of mitochondrial cristae. It does not have the morphological features of apoptosis, such as cell shrinkage, chromatin condensation, skeleton disassembly, or apoptotic vesicle formation (Mou et al., 2019); 2) Unlike apoptosis, ferroptosis occurs without the activation of caspase 3, and the process cannot be reversed by caspase inhibitors; 3) Ferroptosis cannot be interrupted by inhibitors of apoptosis, pyroptosis, or cellular autophagy, but can be inhibited by iron chelators and antioxidants; 4) Ferroptosis can be induced or inhibited through several metabolic pathways (Figure 1), it is characterized by the aggregation of iron and reactive oxygen species (ROS), both of which are thought to be central to ferroptosis, and the inhibition of cystine/glutamate antiporter (cystine/glutamate antiporter system, or System Xc-) and glutathione peroxidase 4 (GPX4) activity, by reducing cystine uptake, depleting glutathione (GSH), and releasing molecules, such as arachidonic acid (Doll and Conrad, 2017); 5) Genetically abnormal expression of several genes can occur, especially of those related to iron metabolism, such as the transferrin receptor (TfR), divalent metal transporter 1 (DMT1), ferritin heavy chain 1 (FTH1), and nuclear receptor coactivator 4 (NCOA4) (Wang et al., 2018). Caspase activation and autophagic lysosome formation are specific markers of apoptosis and autophagy, respectively. Although there are no recognized specific markers for ferroptosis, acyl-coenzyme A synthetase long chain family member 4 (ACSL4) causes the earliest upregulation of adrenaline in non-lethal ferroptosis in various AKI models, which suggests that this molecule is a reliable biomarker for ferroptosis detection. Whether these different modes of cell death can be integrated into a complete regulatory network requires further exploration (Doll et al., 2017).

### Iron Metabolism

Iron homeostasis has an important influence on the incidence of ferroptosis, and iron is present in the circulation as the trivalent iron (Fe^3+^) bound to transferrin. Fe^3+^ is converted to Fe^2+^ by the membrane protein transferrin receptor 1 and divalent m*et al* transport protein 1 and is released into the cytoplasmic labile iron pool (LIP). Excess iron is stored as ferritin, and the ferritin heavy chain (FTH1) has iron oxidase activity that catalyzes the conversion of Fe^2+^ into Fe^3+^, allowing iron to be safely incorporated into the ferritin shell and therefore reduce free iron levels. Hydrogen peroxide reacts with Fe^2+^ to produce hydroxyl radicals with strong oxidizing properties, and this is known as the Fenton reaction. Iron overload causes abnormalities in the mitochondrial oxidative phosphorylation pathway, producing ATP along with large amounts of ROS, oxidizing polyunsaturated fatty acids (PUFA) on cell and organelle membranes, forming lipid peroxides, and directly or indirectly disrupting cellular structure and function. In AKI due to IRI, ROS production is increased during reperfusion and induces ferroptosis in renal tubular epithelial cells. In AKI, plasma catalytic iron concentrations were significantly increased and correlated with extensive injury caused by cisplatin, ischemia-reperfusion, aminoglycosides, rhabdomyolysis, and hemoglobinuria, and both higher plasma catalytic iron levels and lower hepcidin concentrations were associated with increased mortality in patients with AKI (Leaf et al., 2019).

### Amino Acid Metabolism

The glutamate-cystine/antiporter (System Xc-) is a non-sodium-dependent cystine/glutamate reverse transporter protein encoded by the solute carrier family 7 member 11 (SLC7A11), consisting of the catalytic subunit SLC7A11 and the chaperone subunit solute carrier family 3 member 2 (SLC3A2). SLC7A11 is also commonly used to refer to the Xc- system. The Xc-system exchanges glutamate and cystine intracellularly and extracellularly in a 1:1 ratio, and the intracellular cystine is reduced to cysteine. The methionine amino acids generate S-adenosylmethionine under enzymatic action, and cysteine is also produced after demethylation and deadenosylation. Cysteine binds glutamate and glycine to form GSH, which is an intracellular antioxidant that is used in intracellular enzymatic and non-enzymatic antioxidant reactions to maintain hydrogen peroxide levels within the physiological range. GPX4 is a GSH-dependent enzyme with an active center, composed of selenoproteins. Selenoproteins contain selenocysteine that reduces peroxide accumulation by reducing lipid-peroxide (L-OOH) to the corresponding harmless alcohol (L-OH) using GSH and thiol-containing compounds, thereby inhibiting lipoxygenase (LOX) activity and phospholipid/cardiolipid oxidation events. Mevalonate is used as a raw material for the synthesis of isoglutarate pyrophosphate (IPP) from acetyl coenzyme A (CoA), which is an essential signal for the maturation of selenocysteine tRNAs and the synthesis of active GPX4 (Moon et al., 2019). Erastin and sulfasalazine inhibit System Xc- function, resulting in insufficient cystine uptake, decreased intracellular antioxidant capacity, and lipid accumulation, and thereby, inducing cellular ferroptosis. AKI morbidity and mortality were significantly increased in GPX4 knockout mice, and during IRI there was a significant decrease in GSH levels, reduced GPX4 activity, increased iron accumulation and lipid peroxidation, and upregulated expression of proteins and genes associated with iron-sensitivity in kidney tissue (Ma et al., 2021).

### Lipid Metabolism

PUFA contains arachidonic acid (AA) or its derivative adrenergic acid (AdA), and the pentose phosphate pathway directly dehydrogenates and decarboxylates glucose oxidatively to produce nicotinamide adenine dinucleotide phosphate (NADPH) as a reducing agent involved in the synthesis of fatty acids. AA and ACSL4 are esterified into phosphatidylethanolamine (PE), which in turn is formed by LOX through association with recombinant human phosphatidylethanolamine binding protein 1 to form the complex 15-LOX/phosphatidylethanolamine binding protein 1 (PEBP1). This undergoes metamorphic regulation to provide the signal sn2-15-HpETE-PE site that promotes ferroptosis and is ultimately oxidized to phospholipid hydroperoxides (PE-AA/AdA-OOH) and ROS. This is where 15-LO2 is highly expressed in renal tubular epithelial cells, and both 15-LO1 and 15-LO2 are involved in ischemic acute kidney injury (Anthonymuthu et al., 2018). ROS generate destructive hydroxyl radicals through the Fenton reaction, which react rapidly with neighboring molecules or peroxidize with cellular lipid components to generate large amounts of lipid radicals, resulting in thinning of PUFA-rich cell membranes and plasma membranes and irreversible damage to structure and function. Nuclear factor E2-related factor 2 (Nrf2) is considered an important regulator of the antioxidant system, and its downstream target genes are involved in maintaining redox homeostasis. These include GSH synthase, GPX4, and the oxidative stress sensor molecule kelch-like ECH-associated protein 1 (KEAP1), which reduces Nrf2 activity by ubiquitination. In AKI, methylpadoxolone activates Nrf2 by inhibiting KEAP1’s ubiquitin activity to activate Nrf2. This results in improved glomerular filtration rate. Renal lipid peroxidation was found in FA-AKI, and mice pretreated with ferritin-1 had improved renal function and reduced tissue damage (Shimada et al., 2016).

### P53

P53 has become one of the most extensively studied genes since its discovery in 1979. (Liu and Gu, 2021). Apart from its effects on common forms of cell death, p53 is well known for its key role in ferroptosis. (Tang et al., 2019). In the first study to investigate the role of p53 in ferroptosis, we found that p53 promotes ferroptosis by decreasing the expression of SLC7A11 (Jiang et al., 2015). In addition, p53 can regulate ferroptosis and lipid peroxidation by virtue of its target gene spermidine/sper-mine N1-acetyltransferase (SAT1) whose effect is demonstrated by ALOX15 upregulation after SAT1 induction (Gao et al., 2015). Recently, p53 has been discovered to delay the ferroptosis by up-regulating the expression of its downstream target p21 (Tarangelo et al., 2018). Furthermore, in p53-deficient cells, the dipeptidyl-peptidase-4 (DPP4) interacts with NADPH Oxidase 1(NOX1), thus forming a NOX1-DPP4 complex that mediates plasma membrane lipid peroxidation and ferroptosis (Gao et al., 2019). ACSL4 has been reported as both a reliable biomarker and a pivotal contributor to ferroptosis. Recent studies have confirmed that ACSL4 levels are post-transcriptionally downregulated by p53-activated miR-34 (Chang et al., 2007; Jiang et al., 2020). And there is a possibility that the p53/miR-34/ACSL4 axis represses ferroptosis by limiting lipid peroxidation. Controversial results of p53 are quite common in different ferroptosis studies, which may result from “different cell types,” “duality of p53 functions” or “different interventions” (Liu and Gu, 2022).

### Other Mechanisms

Erastin can bind to VDAC2 and VDAC3 in the mitochondrial voltage-dependent anion channel (VDAC) to alter cell membrane permeability and ion selectivity, causing mitochondrial dysfunction and the release of oxidative substances, which ultimately lead to ferroptosis (Yagoda et al., 2007). Heme oxygenase-1 (HO-1) is an important source of intracellular iron, and Adedoyin et al. (2018) found that HO-1 can act as a protective enzyme to inhibit erastin-induced occurrence of ferroptosis in proximal tubular epithelial cells in acute kidney injury. In contrast, in fibrosarcoma cells, Kwon et al. (2015) found that inhibition of erastin-induced ferroptosis was alleviated, and this contradictory result suggests that the exact relationship between HO-1 and ferroptosis requires further exploration. Kinolfin, a class of oral, small molecule, gold-containing compounds for the treatment of RA, was recently found to cause ferroptosis through high doses of inhibition of thioredoxin reductase (TXNRD) activity, which led to lipid peroxidation and ferroptosis, and suggested that TXNRD is a key factor in ferroptosis (Yang et al., 2020). Autophagy-related protein Beclin 1 can form a complex with SLC7A11 to promote ferroptosis, and this process requires AMP-activated protein kinase (AMPK) to mediate phosphorylation of the S90/93/96 sites of Beclin 1 (BECN1) (Song et al., 2018). Bersuker et al. (2019) found that coenzyme Q10 (CoQ10) captures lipid reactive oxygen species through its reduced form, while ferroptosis suppressor protein 1 (FSP1) catalyzes the regeneration of CoQ10 *via* NADH to prevent the release of lipid peroxides, and that FSP/CoQ10 can inhibit ferroptosis independently of the glutathione peroxidase 4 (CPX4) pathway.

As the mechanism of ferroptosis is being studied, many specific ferroptosis inhibitors have been discovered, such as the novel compounds ferrostatin-1 (Fer-1), liproxstatin-1 (Lip-1), and vitamin E, in addition to iron chelators. It is important to clarify the mechanism of ferroptosis and its regulation to provide new research ideas and therapeutic options for diseases related to ferroptosis, and the inhibitors.

## Ferroptosis and Acute Kidney Injury

### Ferroptosis and Rhabdomyolysis Syndrome Resulted in Acute Kidney Injury

The etiology of rhabdomyolysis syndrome is complex, with studies pointing to more than 190 acquired causes and more than 40 genetically related causes. Common causes include strenuous exercise, direct trauma, metabolic myopathy, toxic chemicals, physical or biological agents, and genetic factors (Thévenod et al., 2020). Previous studies suggest that the main mechanisms of rhabdomyolysis-induced AKI are: 1) Accumulation of myoglobin (Mb) in the kidney after massive damage to skeletal muscles that blocks the distal renal tubules. This is the central mechanism leading to renal damage; 2) Leakage of bodily fluids after muscle damage, insufficient blood volume, activation of renin-angiotensin, sympathetic nervous system, release of plasma antidiuretic hormone, other inflammatory mediators, such as endothelin-1 (ET1), tumor necrosis factor-α (TNF-α), which promote vasoconstriction; 3) Nitric oxide in the renal microcirculation is scavenged by Mb resulting in insufficient vasodilator levels. Increased vasoconstrictor and decreased vasodilator levels lead to the constriction of renal arteries, causing inadequate blood supply to the kidneys. Some recent studies suggest that ferroptosis plays a more important role in Mb-induced AKI than previously thought (Tang et al., 2021). In rhabdomyolysis, Mb is released in large amounts beyond the binding capacity of α2-globulin, and Mb is filtered out of the glomerulus into the proximal tubule, where it is eventually broken down into free iron ions and ferrous hemoglobin. Transferrin receptors and divalent metal ion transporters transfer extracellular free iron ions into the cell. Fe^3+^ entering the cell is converted to Fe^2+^, and part of Fe^2+^ is stored in ferritin, while the other part is transported extracellularly by membrane iron transport proteins to be rebound by Hb or Mb (Tang et al., 2021). If there were too much Mb in the renal tubules, it would lead to Fe^2+^ overload in the renal tubular cells in the presence of insufficient intracellular ATP, hypotension, and hypoperfusion. Overloaded Fe^2+^ will then induce direct damage to proximal tubular lipid peroxidation through the Fenton reaction, leading to acute tubular necrosis or renal failure (Bosch et al., 2009; Zeng and Tomlinson, 2021), where ferritin heavy chain 1 (FTH1) is of importance (Xie et al., 2016a). Zarjou et al. (2013) found that FTH1 knockout mice had higher mortality rates and more severe kidney injury than wild-type mice in a rhabdomyolysis-induced AKI model, which shows the protective effect of FTH1 against tubular injury and the role of iron ions in AKI. A study by Guerrero-Hue et al. (2019) found that Fer-1, a small molecule inhibitor of ferroptosis, could inhibit Mb-induced AKI, a finding that demonstrates the key role played by ferroptosis in this process. Skouta et al. (Tang et al., 2021) also found that Fer-1 effectively inhibited hydroxyquinoline and ferrous ammonium sulfate-induced cell necrosis in a rhabdomyolysis-induced *in vitro* ferroptosis model. In summary, the literature show that ferroptosis is one of the important mechanisms causing rhabdomyolysis-induced AKI.

### Ferroptosis and Acute Kidney Injury due to Ischemia Reperfusion Injury

Ferroptosis and AKI due to ischemia reperfusion injury (IRI) is a condition in which the degree of tissue damage increases rapidly after restoration of blood flow to cells that have suffered a period of ischemia, resulting in a clinical condition called reperfusion syndrome. This process exacerbates tissue injury by initiating an inflammatory cascade of responses. The cascade mainly covers ROS, cytokines, chemokines, and leukocyte activation. (Nieuwenhuijs-Moeke et al., 2020; Messerer et al., 2021). IRI is also an important factor in contributing to AKI. The main pathophysiological mechanisms of renal IRI include inflammation, oxidative stress and lipid peroxidation, mitochondrial dysfunction, and activation of the renin-angiotensin system (Franzin et al., 2021). A recent study suggests that ferroptosis may be one of the main drivers of renal IRI (Tonnus and Linkermann, 2017). In a mouse model of IRI, application of ferroptosis small molecule inhibitors protected mice from AKI and other organ damage (Tonnus et al., 2021). In clinical practice, renal IRI is usually the leading cause of AKI after cardiac surgery. The pathogenesis of postoperative AKI in the heart is complex and multifactorial, and these mechanisms of injury might play different roles at different times and may also act synergistically, with the release of free iron being a key part (Han et al., 2021). Due to the prolonged exposure of blood cells to the mechanical extracorporeal circulation system during various manipulations, such surgical procedures may lead to the destruction of red blood cells, resulting in the entry of free Hb into the circulation (van Swelm et al., 2020). It has been shown that free Hb levels in the blood increase to several times the physiological level during extracorporeal circulation and remain so until several hours after the procedure (Lee et al., 2021). The role of Hb in the induction of AKI is similar to that of Mb, with tubular cell iron overload being the main cause of eventual acute renal failure or tubular necrosis (Tang et al., 2021); studies have shown that free Hb levels are a significant independent risk factor for renal impairment after cardiac surgery (Leaf et al., 2019). Hemodynamic instability might occur during the transition from complete extracorporeal circulatory support to the patient’s own circulation, and this state of hemodynamic instability likely leads to systemic hypoperfusion, particularly ischemia and hypoxia of the kidneys (Haase et al., 2010). During ischemia, free iron levels in the kidney are increased by lower pH and dissociation of protein-bound iron. An increase in free iron might activate cellular ferroptosis, ultimately leading to tubular necrosis (van Swelm et al., 2020). Choi et al. (2019) proposed that lower intraoperative levels of iron-binding protein indirectly reflect impaired processing of catalytic iron by the body during extracorporeal circulation, leading to renal injury. This result emphasizes the importance of iron homeostasis in ischemia-reperfusion injury and suggests that iron homeostasis is a potential therapeutic target in kidney injury associated with cardiac surgery or in AKI induced by ischemia-reperfusion (Choi et al., 2019). In addition, studies have found that mechanical ventilation is an independent risk factor for the development of AKI after cardiac surgery. In an IRI mouse model, prolonged mechanical ventilation led to a gradual decrease in GPX4 levels, increased renal lipid peroxidation, and a gradual decrease in blood GSH and renal homogenate GSH levels. This suggests that prolonged mechanical ventilation leads to blood GSH depletion and induces renal ferroptosis *via* the GSH⁃GPX4 axis (Zhou et al., 2020a). In a model of hypoxic injury to renal tubular epithelial cells, application of small molecule inhibitors of ferroptosis effectively attenuated hypoxic damage to renal tubular epithelial cells, which suggests that ferroptosis may be an earlier mode of cell death in hypoxic renal tubular epithelial cells and that hypoxia plays a critical role in the development of ischemia-reperfusion AKI (Li et al., 2021a). Reperfusion injury to other organs during extracorporeal circulation may also bring additional iron to the kidney, and iron released from the upstream necrotic kidney unit might further descend to the next kidney unit, further exacerbating oxidative stress and inducing tissue damage (Martines et al., 2013). Linkermann et al. (Tonnus et al., 2021) found that ferroptosis is a key mechanism for sequential renal tubular cell death after ischemic AKI, and that triggering ferroptosis by inhibiting the cysteine glutamate transporter and applying the ferroptosis activator erastin to reduce intracellular GSH could cause proximal tubular cell cascade cell death. The use of Fer-1 not only inhibited ferroptosis *in vitro*, but also attenuated ischemia-reperfusion injury in mouse kidneys. Therefore, it can be inferred that ferroptosis is one of the important mechanisms of ischemia-reperfusion AKI. Huang et al. (2019) inhibited the expression of augmenter of liver regeneration (ALR) in an *in vitro* model of IRI-induced AKI and found that the level of cellular ferroptosis was increased, along with an increase in ROS and significant mitochondrial damage. In addition, the inhibition of System Xc⁃ with erastin promoted cellular ferroptosis and silenced ALR expression, which suggests that ischemia-reperfusion-induced AKI is mediated by ALR and that this process is associated with the glutathione⁃glutathione peroxidase (GSH⁃GPx) system. It was recently shown that IRI induced the upregulation of miR-182-5p and miR378a-3p and further downregulated GPX4 and SLC7A11, which also induced ferroptosis in AKI (Ding et al., 2020).

### Ferroptosis and Drug-Induced Acute Kidney Injury

Cisplatin is an important chemotherapeutic agent for the treatment of many solid tumors, but its clinical application is limited by serious adverse effects, especially nephrotoxicity. Baliga et al. (Sharma and Leaf, 2019) reported in 1998 on a cisplatin-induced cytotoxicity *in vitro* model and a cisplatin-induced acute renal failure *in vivo* model, which showed that iron was detectable in bleomycin released into the medium after exposure to cisplatin, which, a significant increase in iron that could catalyze free radical reactions. They also found that iron chelators significantly reduced cisplatin induced cytotoxicity, suggesting an important role for iron, but the exact mechanism could not be elucidated. Ferritin plays a central role in iron metabolism, and it was found that in the proximal tubule, FTH1 knockout mice had more severe renal injury after cisplatin administration than the control group, which is a finding that underscored the protective role of FTH1 in AKI (van Swelm et al., 2020). Inositol dioxygenase is a proximal renal tubular enzyme, whose overexpression was found by Deng et al. (2019) to exacerbate cisplatin-induced redox damage in cells with AKI. They also found that it promotes ferroptosis through ferritin phagocytosis and lipid peroxidation, and that it might also inhibit GPX4 activity and intracellular GSH concentration by downregulating the “ferroptosis termination system.” In rodents, folic acid could lead to the development of AKI, and certain doses of folic acid could form crystals in the kidney lumen, and high doses could also be directly toxic to the renal tubular epithelium. Martin⁃Sanchez et al. (2017) confirmed the presence of lipid peroxidation and GSH downregulation, which are typical features of ferroptosis, in a folic acid-induced AKI mouse model. In addition, the ferroptosis inhibitor Fer-1 reduced oxidative stress, decreased tubular cell death, and attenuated tissue injury by inhibiting the upregulation of chemokines and cytokines, such as interleukin 33 (IL-33), and by inhibiting macrophage infiltration and downregulating the protective factor Klotho. Caspase inhibitors, however, had no nephroprotective effect (Martin-Sanchez et al., 2017). It was also found that receptor⁃interacting protein kinase 3 (RIPK3) and mixed lineage domain⁃like protein kinase (MLKL) were inhibited. However, the use of RIPK1 inhibitors or RIPK3 and MLKL gene defects did not prevent kidney injury, which suggests that ferroptosis was the predominant cell death pathway in folic acid-induced AKI (Martin-Sanchez et al., 2017). One recent study from China found that higher p53 activation in tubular cells of folic acid-induced AKI model, while Α-lipoic acid (ALA) supplementation blocked the activity of p53 and inhibit ferroptosis (Li et al., 2021b). Thus, p53-mediated ferroptosis in tubular epithelial cell may be a target of treatment of AKI. FG⁃4592 is an inhibitor of hypoxia⁃inducible factor (HIF) precursor hydroxylase, and Li et al. (2020) recently found in a folic acid-induced AKI model that renal function was significantly improved in FG⁃4592 pretreated mice, while the levels of iron, malondialdehyde, and 4-hydroxynonenal in tissues were reduced. In addition, there was upregulation of HIF-1α expression, activation of nuclear factor E2 related factor 2 (Nrf2), and high expression of downstream proteins, including hemeoxygenase 1 (HO1), GPX4, System Xc⁃, and membrane iron transport protein. Further signaling pathway studies suggested that Nrf2 activation is regulated by protein kinase B/glycogen synthase kinase-3β (PKB/GSK⁃3β), which suggests that PKB/GSK⁃3β is a potential therapeutic target for ferroptosis.

### Others

Increased iron accumulation and lipid peroxidation in the renal tissue of severe acute pancreatitis rats showed that these changes were accompanied by decreased GPX4 activity and up-regulation of ferroptosis-related proteins and genes. This demonstrates that ferroptosis is associated with severe acute pancreatitis-induced AKI, and lipase I (LIPI) could inhibit ferroptosis, and both reduce renal damage and improve renal function (Ma et al., 2021).

## Treatment of Ferroptosis in Acute Kidney Injury

**FIGURE 2:**
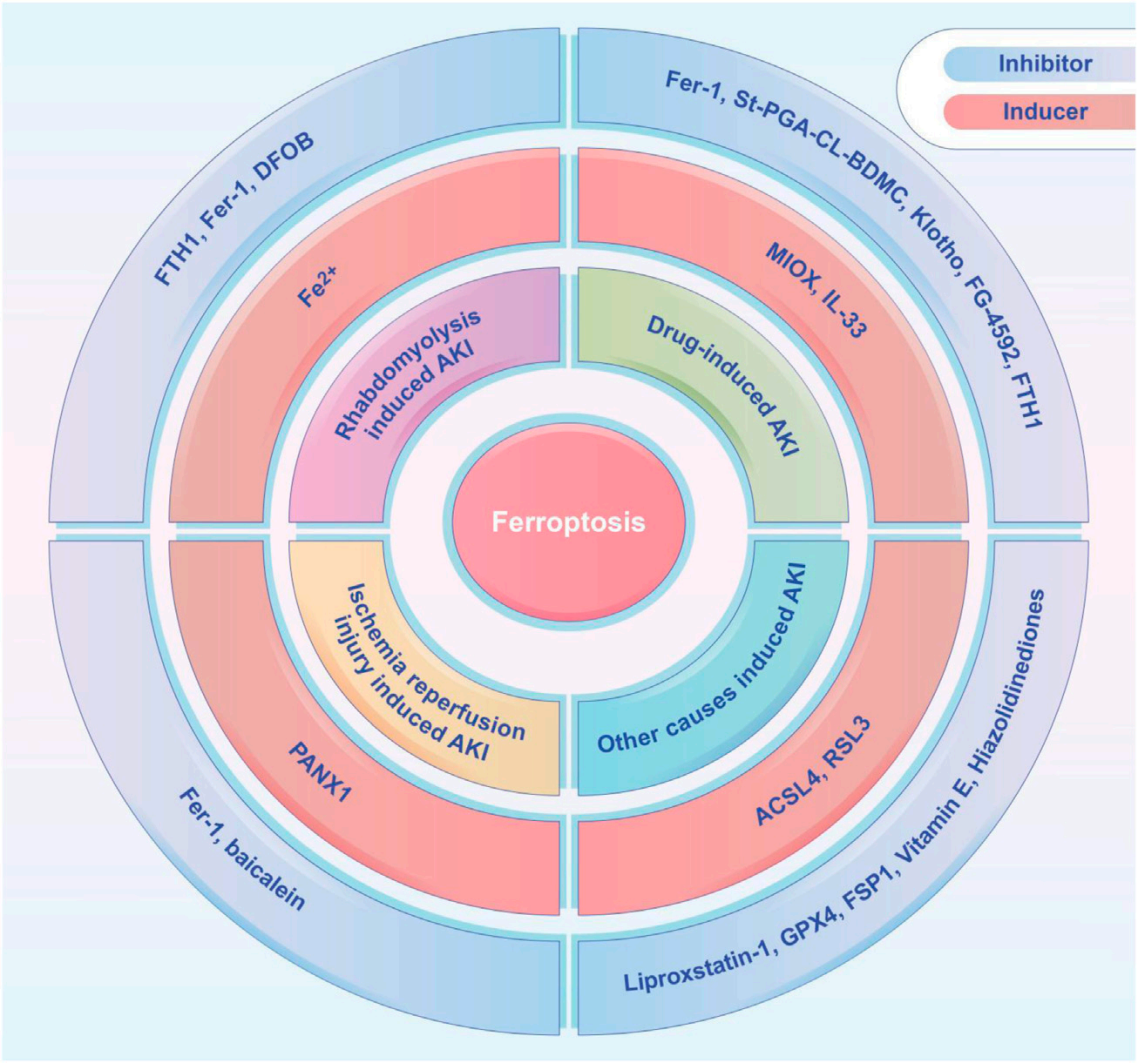
The ferroptosis inducers and inhibitors tested in animal models of AKI, including rhabdomyolysis induced AKI, drug-induced AKI, ischemia-reperfusion induced AKI, and other causes induced AKI. ACSL4, acyl-coenzyme A synthetase long chain family member 4; DFOB, desferrioxamine B; FTH1, ferritin heavy chain 1; Fer-1, ferrostatin-1; FG-4592, roxadustat; FSP1, ferroptosis suppressor protein 1; GPX4, glutathione peroxidase 4; IL-33, interleukin 33; MIOX, myo-inositol oxygenase; PANX1, pannexin1; St-PGA-CL-BDMC, star-shaped polyglutamate conjugate of bisdemethoxycurcumin.

Recent studies demonstrated the importance of ferroptosis in AKI and many studies have shown that ferroptosis can be successfully modulated in different kinds of AKI. A variety of ferroptosis inducers and inhibitors have been administrated in those studies (Figure 2). Several studies have shown that ferroptosis is a promising therapeutic target, especially in diseases dominated by tubular necrosis (Linkermann, 2016). GPX4 is an antioxidant enzyme that catalyzes the reduction of lipid peroxides at the expense of reducing GSH, and thereby preventing oxidative damage and ferroptosis. If GPX4 is absent, it will trigger the accumulation of lipid peroxidation products, which eventually leads to the death of mice due to acute renal failure (Demuynck et al., 2021). Small molecule ferroptosis inhibitors (lipoxygenases), which completely block lipid peroxidation, were able to increase the survival rate of GPX4-deficient mice by 35% (Kang et al., 2021). A novel ferroptosis inhibitor1 that is independent of the classical GPX4 signaling pathway was also recently identified. It uses reduced NADPH to reduce CoQ10 to CoQ10 H2 in the cell membrane which reducing cell membrane lipid peroxidation and thereby inhibiting ferroptosis (Bersuker et al., 2019; Doll et al., 2019). Another study found that thiazolidinediones (e.g., rosiglitazone) reduced mortality in GPX4-deficient mice by inhibiting ACSL4, and demonstrated their potential benefit for AKI (Doll et al., 2017; Kagan et al., 2017). Fer-1 can prevent membrane lipid damage through redox reactions, thereby inhibiting cellular ferroptosis (Xie et al., 2016b; Martin-Sanchez et al., 2017; Zilka et al., 2017; Zhou et al., 2020b). Linkermann et al. (Tonnus et al., 2021) found that the use of Fer-1 not only blocked erastin-induced ferroptosis *in vitro*, but also prevented renal IRI in mice. It was also found that desferrioxamine could be used to obtain the less cytotoxic compound 3-Hydroxy-Adamant-1-yl by binding to iron adamantine derivatives, which attenuated AKI-causing rhabdomyolysis in rats by inhibiting lipid peroxidation, reducing the ferrous form of Mb, and inhibiting ferroptosis (Groebler et al., 2011; Yu et al., 2017). Pannexin1 (Panx1) is an ATP release pathway family protein. Su et al. (2019) found in a mouse model of ischemia-reperfusion-induced AKI that silencing Panx1 expression significantly attenuated lipid peroxidation and iron accumulation, mainly through mitogen-activated protein kinase/extracellular signal-regulated kinase (MAPK/ERK) signaling. MAPK/ERK signaling activated ferritin and regulated NCAO4-mediated iron phagocytosis and HO1 expression, which provided potential therapeutic targets for AKI management (Su et al., 2019). Deferiprone has been used in patients with high ferrous load from repeated transfusions, but there have been no studies related to the application of deferiprone for the prevention or treatment of AKI due to rhabdomyolysis (Szymonik et al., 2021). Baicalein, an important active ingredient in the Chinese herb Scutellaria baicalensis, attenuated myocardial ischemia-reperfusion-induced AKI by regulating the B-cell lymphoma-2 (BCL2), extracellular signal-regulated kinase 1/2, and BCL2 associated X (BAX) activation of protein kinase B (PKB) (Lai et al., 2016; Szymonik et al., 2021). It has been shown that baicalein can also enhance cellular resistance to ferroptosis by limiting iron accumulation and lipid peroxidation in cells, which makes it a promising therapeutic agent for ferroptosis-related tissue damage (Xie et al., 2016b).

In a mouse model of folic acid-induced AKI, Córdoba-David et al. (2020) proposed that stellate polyglutamate⁃curcumin coupling inhibited nuclear factor kappa B (NF-κB) activation and downregulated ferroptosis marker expression, while preserving the renal expression of Klotho, ultimately exerting a reno-protective effect. In addition, rhabdomyolysis-induced renal dysfunction and histological damage could also be reduced by curcumin treatment, with the involvement of HO1 (Guerrero-Hue et al., 2019). Li et al. (2021b) confirmed that ALA also could be used as an anti-ferroptosis agent to reduce iron overload in folic acid-induced AKI through upregulation of Ferritin and ferroportin (Fpn). Additionally, LA has the ability to increase the expression of system xCT, thus increasing the synthesis of GSH and enhancing the activity of GPX4. Roxadustat, an inhibitor of the hypoxia-inducible factor prolyl hydroxylase, is protective against folic acid-induced AKI, and studies have shown improved renal function, inhibition of iron accumulation and lipid peroxidation, and increased levels of antioxidant enzymes and GSH in pretreated mice as compared to untreated mice (Li et al., 2020). Mishima et al. (2020) found in a mouse model of cisplatin-induced AKI that cytochrome P450 (CYP450) substrates, such as isoproterenol and rifampin, attenuated tissue damage and cell death, by scavenging lipid peroxidation radicals. However, due to non-specific ferroptosis markers, multiple cell death pathways might exist in the injury model, and the inhibitory effect of drugs on ferroptosis was not directly confirmed. Some studies have tentatively shown that vitamin D receptor (VDR) activation is protective against cisplatin-induced AKI, and that paricalcitol reduces lipid peroxidation, 4-hydroxynonenal, and malondialdehyde, functionally and histologically attenuating cisplatin-induced AKI (Mishima et al., 2020).

## Conclusion

Ferroptosis is a recently discovered, iron-dependent, non-apoptotic cell death mechanism. This paper outlines the mechanisms underlying its incidence, its characteristics, and its general preliminary understanding. In AKI, it has been possible to clarify that ferroptosis is one of the important causes of cell death. The application of small molecule ferroptosis inhibitors to inhibit ferroptosis is expected to be a new strategy in the treatment of AKI. It is applied in AKI particularly in cases of rhabdomyolysis or ischemia-reperfusion AKI, which is mainly caused by heme and non-matrix iron. Although the majority of these studies targeting small molecule ferroptosis inhibitors have been conducted in mice models of AKI or *in vitro* experiments, we believe that an in-depth exploration of the role played by ferroptosis in the process of AKI and the rational use of ferroptosis in the regulation of AKI would provide new perspectives and new strategies for AKI treatment.
